# Corticosterone response to gestational stress and postpartum memory function in mice

**DOI:** 10.1371/journal.pone.0180306

**Published:** 2017-07-10

**Authors:** Zahra Jafari, Jogender Mehla, Navvab Afrashteh, Bryan E. Kolb, Majid H. Mohajerani

**Affiliations:** 1 Department of Neuroscience, Canadian Centre for Behavioural Neuroscience (CCBN), University of Lethbridge, Lethbridge, AB, Canada; 2 School of Rehabilitation Sciences, Iran University of Medical Science (IUMS), Tehran, Iran; Universite de Rennes 1, FRANCE

## Abstract

Maternal stress is a common adversity during pregnancy. Gestational corticosterone alternations are thought to contribute to the etiology of postpartum behavioral disturbances. However, the impact of stress during pregnancy, in particular noise exposure, on gestational corticosterone fluctuations and spatial cognition in postpartum mice has not been fully understood yet. We hypothesized that noise exposure during pregnancy negatively affects gestational corticosterone levels and postpartum memory function in the dams similar to the physical stressors. Pregnant C57BL/6 mice were randomly assigned to either one of two stress conditions or a control condition. The noise stress (NS) was induced by presenting a loud intermittent 3000 Hz frequency on gestational days (GDs) 12, 14, and 16 for 24 hours, whereas the physical stress (PS) consisted of restraint and exposure to an elevated platform on GDs 12–16. Plasma corticosterone level was collected on GDs 11 and 17, and Morris water task (MWT) was carried out 30 days after parturition. Compared to the control group, the level of corticosterone in the stressed groups was significantly increased on GD17 relative to GD11. Significantly longer swim time and lower swim speed were observed in both stressed groups relative to the control group. Probe time was significantly shorter in the NS group than the other groups. The delta corticosterone level was significantly correlated with the swim time as well as the probe time in the three groups. Given the results, the adverse effects of gestational noise exposure on the hypothalamic pituitary-adrenal (HPA) axis activation and postpartum spatial learning and memory function were as large as/ or a bit stronger than the physical stresses. The findings suggest the significance of conservation against loud noise exposure in daily living, as well as need to further notice to the different aspects of gestational stress in mothers’ behavior like offspring.

## Introduction

Stress, as an inherent component of modern life, has a major contribution in shaping the brain and behavior [1, 2]. The term “stress” has different definitions in the literature but in laboratory animal studies, it usually refers to the impact of an event on the organism and the organism’s response to it. Stress causes distress and produces emotional and cognitive changes in the pregnant animal and in her offspring subsequent to adaptive physiological responses and the release of hormones [3]. Exposure to environmental stresses during gestation, such as social, physical, or mental pressures, increases the risks of psychopathology in offspring; these adverse effects of environmental stress are well studied [4, 5]. The detrimental effect of gestational stress on parental care and maternal behavior are also very well investigated [6, 7]. However, a few studies examined the influence of stress during gestation on cognitive performance in mice postpartum [8, 9].

Reproductive experience has long-lasting effects in both rodent and human brains [10]. Rodent research has shown pregnancy, motherhood, and attendant offspring care cause changes in the brain and behavior; and, this has an impact on cognition, affect, and response to stress [11]. Motherhood has also been shown to augment hippocampal-dependent learning and memory [12, 13]. Thus, higher resilience to stress, decreased anxiety, and enhanced memory performance have been shown in primiparous (one pregnancy and bout of rearing) and multiparous (multiple pregnancies and bouts of rearing) rats compared with nulliparous rats without pregnancy experience [14]. The behavioural and cognitive changes last much longer after pregnancy, persisting even into old age [11, 14–16]. Interestingly, in a study of the acquisition of the Morris water task (MWT) by primiparous and nulliparous female rats exposed to gestational restraint stress, both groups of animals were impaired relative to controls; this suggests gestational stress can counteract the advantages of reproduction on postpartum learning and memory performance [14]. Stress-related hormones, in particular glucocorticoids, which adversely impair cognitive functions [5, 14, 17, 18], are presumed to play a significant role in the gestational stress effects. Whereas pregnancy itself highly increases secretion of the corticosterone [17], maternal stress markedly intensifies the corticosterone level leading to an impairment in hippocampal function in mothers and offspring [14, 17]. In spite of the importance of the gestational stress effects on hippocampal function in the mothers, to date there has been little research on it [17].

Noise, which can be defined as an unwanted unpleasant auditory stimulus, is a major environmental pollutant that can produce significant biochemical and neural changes in animals as well as in humans [17]. Noise can lead to negative health effects if the exposure time is prolonged and/or exceeds certain levels. Studies have frequently indicated that high levels of noise are markedly stressful and significantly enhance release of stress hormones, i.e., glucocorticoids, leading to impairments in learning and memory [17, 19, 20]. While evidence of an adverse impact of environmental noise exposure on public health (annoyance, disturbed sleep, increased the occurrence of hypertension and cardiovascular disease, and impaired cognitive performance) is growing [19, 21], noise exposure has been less investigated as a gestational stressor [9]. Thus, among the diverse types of gestational stressors, restraint is the most commonly used and noise is the least used stressor [4]. It is well known that the exposure to various environmental stresses, including noise [17, 22–24], causes morphological and non-morphological changes in the hippocampus and impacts hippocampal neurogenesis; a brain process which has been shown to continue until aging in a myriad of mammalian species [11, 25]. Little evidence, however, is available regarding the effects of gestational noise exposure on hippocampal plasticity and memory function in postpartum rodents.

The present study aimed to investigate the effects of two types of stressors during pregnancy, noise stress and physical stress, on gestational corticosterone levels and postpartum spatial learning and memory function in mice. Given the negative effects of loud noise levels on release of stress hormones and cognitive performance [17, 19], we hypothesized that gestational noise exposure strongly activates the hypothalamic–pituitary–adrenal (HPA) axis and adversely affects spatial learning and memory function in the postpartum dams as large as the physical stressors [5, 26], i.e., restraint combined with placement on an exposed elevated platform in the present study.

## Material and methods

### Animals

All experiments were carried out in accordance with the Canadian Council of Animal Care and approved by the University of Lethbridge Animal Care Committee. All animals were given access to food and water ad libitum and were maintained on a 12:12-h light:dark cycle in a temperature-controlled breeding room (21°C) with less than 66±2 dB room noise level. Thirty female C57BL/6 mice between 8 to 11 weeks of age were individually mated with thirty male C57BL/6 mice in standard shoe-box cages at 4:00 pm. For recording of gestational length, the female mice were assessed three hours later at 7:00 pm and the next morning for breeding signs such as sperm plug and red/swollen vaginal opening [17]. If a plug or sperm was present, the female was considered possibly pregnant and removed from the male till the pregnancy confirmed, and then housed with 2–3 females by 1–2 days before parturition. Once a female was left with a male overnight, she was not paired with a male again until the lack of pregnancy was confirmed. The weight gain of the female mice was followed every day to confirmed pregnancy. On the gestational day (GD) 11, a weight gain of at least 3.5 g usually signifies conception has occurred. This method allows a determination of the length of gestation with a 0.5-day precision. When the pups were born, the dams were kept individually with the litters.

### Experimental design

Pregnant mice were randomly assigned to three groups consisting of two stress groups and one control group. The time range of the most reported gestational stress exposures is on GDs 14–21 [27–30]. We exposed animals to stress on GDs 12–16 because the corticogenesis process occurs from embryonic day 10 to 17 in mice, and the layer II/III, IV, and V mainly develop during GDs 12–16 [1, 31]. This timeframe also corresponds to the third trimester in human pregnancy when substantial neural development occurs [32].

#### Physical stress (PS) group

Two stressors, restraint and elevated platform (EP), were applied daily from GDs 12 through 16. For restraint, mice (n = 10) were maintained in a transparent Plexiglas container (5 cm inner diameter), 20 minutes per day at 10:00 am. The container maintained the mice in a standing position without compression of the body [33, 34]. For the EP stressor, each mouse was placed on an elevated platform (1m height, 21×21 cm), 30 minutes twice a day at 9:00 am and 3:00 pm [35, 36].

#### Noise stress (NS) group

On gestational days (GDs) 12, 14, and 16, a female pregnant mouse was transferred into a standard cage and moved to a sound chamber. A speaker, which emitted an intermittent 3000 Hz frequency sound of 90 dB [37–39] for 1 sec duration and 15 sec inter-stimulus interval (ISI) [40], was placed in the cage. The sound pressure level was measured daily inside the cage without an animal (Tektronix RM3000, Digital Phosphor Oscilloscope). The mice (n = 10) were exposed to the NS for 24 hrs starting at 8:00 am. Similar protocol was previously used in rats, including a low-frequency sound of 300 Hz for 1 sec in the intervals of 15 sec during 24 hrs. [40]. Here we used a 3000 Hz frequency tone, since (a) is audible by mice [41, 42] and (b) is relatively similar to environmental and traffic noises which are largely made up of low to mid frequency tones [43, 44]. The intensity of NS exposure used in previous studies was also between 95 to 130 dB [17, 37–39]. We applied an intermittent stimulus intensity (90 dB) to prevent noise-induced hearing loss. In addition, 24 hrs rest after every stress exposure will provide enough time for to recover from possible temporary threshold shifts [45].

#### Control group

There were two sets of control animals: one served as a control for NS dams and another was a control for PS dams. In NS control group, pregnant mice (n = 5) on GDs 12, 14, and 16 were individually transferred into a standard cage and moved to a sound chamber. A silent speaker was placed in the cage. The mice were left undisturbed for 24 hrs starting at 8:00 am. In PS control group, pregnant mice (n = 5) were removed daily from the home cage for 20 or 30 minutes (depending on the type of stressor) during GDs 12–16, transferred to the same testing room for the PS, left undisturbed, and then returned to their home cages. In the control group, no stress was given.

### Plasma corticosterone assay

The blood sampling procedure was performed at 7:30 to 8:30 am [46] on GDs 11 and 17, i.e., one day before starting stress regimen, and a day after finishing the stress exposure in the stressed groups. The blood samples were also collected from the control groups without stress using the same schedules. A commercially available enzyme-linked immunosorbent assay (ELISA) kit from Abcam (ab108821) was used to quantify the levels of corticosterone in the plasma. Approximately 0.1 ml of submandibular blood [47] was collected in heparin-coated tubes. These tubes were centrifuged at 6000 rpm at 4°C for 15 min [48] to collect the plasma [39]. Collected plasma samples then were stored at −80°C until further analysis. The assay was carried out as per the manufacturer’s instructions. All reagents were brought to room temperature before starting the assay. The assay was performed at room temperature (20–30°C). The plasma samples were diluted 1:100 into 1X diluent as suggested in the ELISA kit. Briefly, 25 μl of standards or samples were added to the microplate wells. Twenty-five μl of biotinylated corticosterone was then added immediately to each well (on top of the sample or standard). The wells were tightly sealed with Parafilm and incubated for two hours at room temperature. After two hrs of incubation, the microplate was washed six times with 300 μl of wash buffer using plate washer (ELX50 BioTek®). The plate then was inverted and tapped 4–5 times on absorbent paper towel to completely remove the liquid. Fifty μl of streptavidin-peroxidase conjugate then was added to each well and incubated for 30 minutes. After incubation, the microplate was again washed six times with 300 μl of wash buffer using a plate washer. The plate then was tapped 4–5 times on absorbent paper towel to completely remove the liquid. After the liquid removal 50 μl of chromogen substrate was added per well and incubated for 30 minutes. Finally, 50 μl of stop solution was added to each well, leading to the color changing from blue to yellow. The optical density of corticosterone was read at 450 nm wavelength using a microplate reader (Synergy HT BioTek®) within 15 min after addition of stop solution. The concentration of corticosterone in samples was calculated using KC4 Bio-Tek® Microplate Data Collection and Analysis software. To reduce intra-plate variability, the coefficient of variation (CV) for all samples was determined using the same standards and controls across all plates, and only samples with a CV less than 10 percent were included in the analysis. The corticosterone concentration in plasma samples was expressed in ng/ml [17, 46, 49–52].

### Behavioral assessment

The MWT (acquisition and probe tests) was performed for the dams 30-days after parturition. Weaning occurred on postnatal days 21–23. The water task consisted of a pool (153 cm diameter) filled with water (23–25 ±1°C) up to a level of ~15 cm from the top edge of the tank. The water was made opaque by non-toxic white tempura paint. The pool was located in a room rich with distal cues, which remained unobstructed throughout the duration of the experiment [53]. During all hidden platform trials, the platform was submerged ~1.0 cm under the surface of the water. Each trial began with the mouse being placed in the pool in a pseudo-random sequence at one of the four cardinal compass positions around the perimeter of the pool. The tank was divided into four quadrants, 1, 2, 3 and 4 using software, with starting points at north, west, east and south. The starting positions of the mice were located at the intersection of the quadrants, 4–5 cm away from the edge of the tank. Animals were trained with 4 trials per day for 8 consecutive days under regular room light (Water2100 Software vs.7, 2008). The acquisition trial was started by placing a mouse facing the wall of the tank at one of the 4 starting locations. Testing was stopped after the mouse reached the platform or, if the mouse did not find the platform, at the 60 second trial time limit. If a mouse found the platform within this 60 sec period, it was allowed to remain on the platform for five additional seconds. If it did not find the platform during the selected time, it was placed onto the platform for 15 sec by the experimenter before being returned to her home cage. Data were recorded using an automated tracking system (HVS Image Hampton, U.K.). Following each swim trial, the animal was dried with a soft fabric cloth and placed back into the home cage where it was allowed to rest for at least 5 min before the start of the next swim trial. The swim time (sec), swim speed (m/s), and swim distance (m) were calculated for analysis [54].

The probe trial was carried out on the ninth day, in which the platform was removed and each mouse was allowed to swim freely for 60 sec. In order to preclude the possible impact of working memory on retention [55] this trial was performed 24 hours after the last platform trial. The time spent in the quadrant where the platform had been located was measured.

### Statistical analysis

All statistical analyses were done using SPSS Statistics 24.0 using an alpha level of 0.05. We used the Kolmogorov–Smirnov test for normally distributed data. A Multivariate analysis of variance (MANOVA) was used to test for differences between the three studied groups: for different parameters of the MWT, the plasma corticosterone level on GD11 and GD17, and the delta corticosterone level (the difference in the corticosterone levels between GD11 and GD17). To make clear the possible effect of swim speed on the swim time we used a multivariate analysis of covariance (MANCOVA) to compare the three groups in swim time where swim speed was considered as a covariate. A repeated measures ANOVA was used to compare the eight days of training in the MWT, as well as the corticosterone levels on GDs 11 and 17 in each group. The Tukey post-hoc test was performed for multiple comparisons of group means in each measurement. The Pearson correlation coefficient test was used to determine the relationship between variables.

## Results

The ages of the female mice did not differ across the groups (F = 0.697, p = 0.507). Since no significant differences were observed between the two control groups in any of the measures used in this study (p>0.05), the results of the control groups were pooled together.

### Plasma corticosterone levels

#### Corticosterone levels comparison between GD11 and GD17

**Fig 1:**
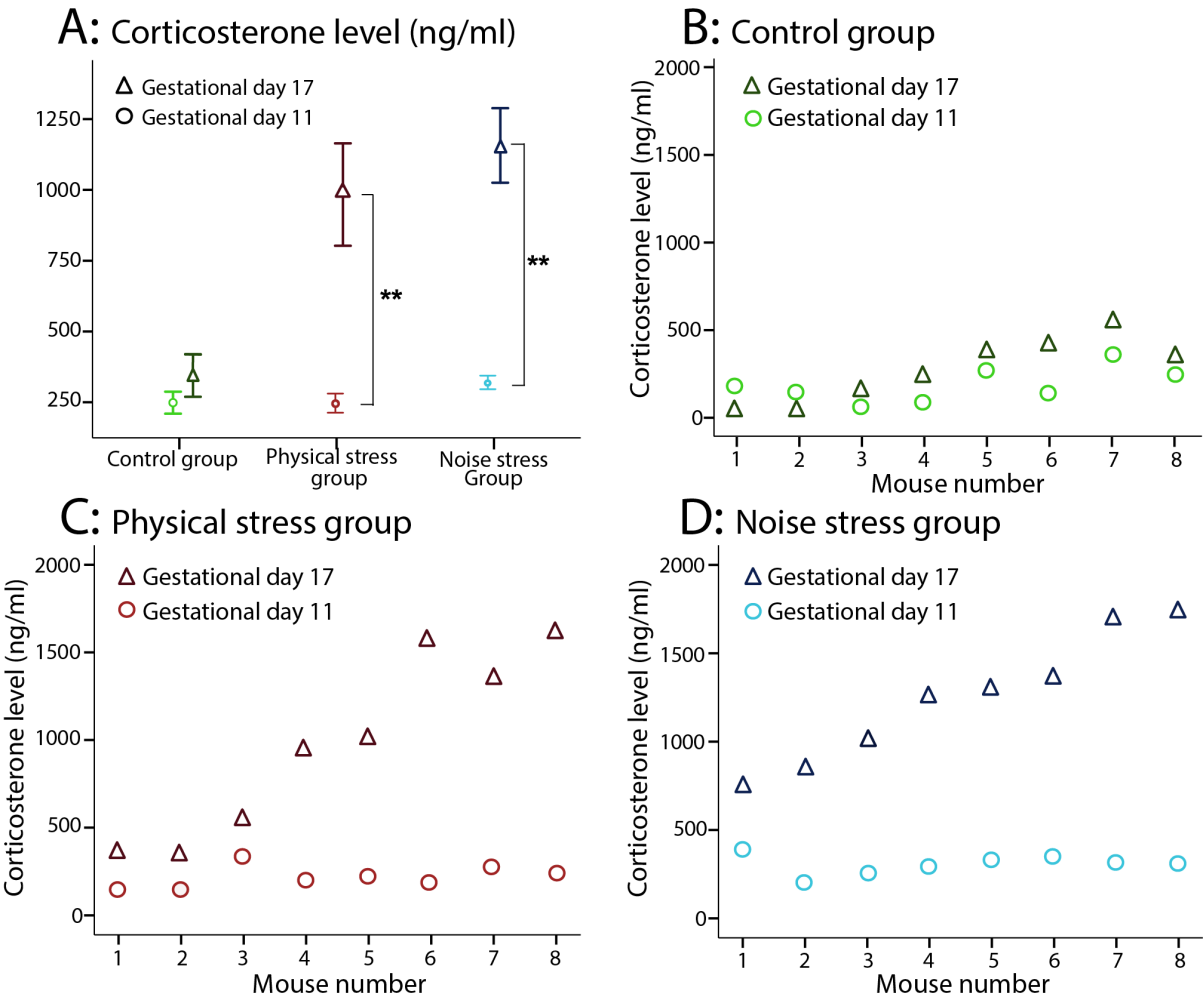
The corticosterone levels (ng/ml): A) A significant increase in corticosterone levels on gestational day (GD) 17 than GD11 in both stressed groups compared with the control group (○: GD11, Δ: GD17). Results reported as mean ± S.E.M. B) No significant difference in corticosterone levels between GD11 and GD17 in the control group. C) A significant difference in corticosterone levels between GD11 and GD17 in the PS group. D) A significant difference in corticosterone levels between GD11 and GD17 in the NS group. N = 8 in the three groups. Asterisks indicate *p<0.05 or **p<0.01.

Blood collection was conducted on GDs 11 and 17 in the three groups. Table 1 compares the two corticosterone measures (ng/ml) across groups. No significant difference was found between the two plasma corticosterone measures in the control group. But in both stressed groups, particularly the NS group (Fig 1A), the corticosterone level was significantly higher on GD17 compared with GD11.

**Table 1:** Comparison between corticosterone levels on gestational days 11 and 17 in every group.

Corticosterone levels (ng/ml)	Mean difference (delta corticosterone)	F	p	η^2^	Power
Control group	95.91	2.129	0.188	0.233	0.244
Physical stress group	736.64	17.933	**0.004**	0.719	0.950
Noise stress group	836.51	44.876	**<0.001**	0.865	1.000

η^2^ = estimates of effect size

#### Corticosterone level comparison among the groups

As Table 2 shows, no significant difference was observed in the corticosterone levels (ng/ml) among the three groups on GD11. However, the corticosterone levels of the stressed groups were significantly higher compared with the control group on GD17. Similarly, the delta corticosterone levels were significantly higher in both stressed groups than the control group (Fig 1A, Table 2). Fig 1B, 1C and 1D demonstrate the corticosterone levels of each animal on GD11 and GD17 in control (n = 8), PS (n = 8), and NS groups (n = 8), respectively. The delta corticosterone level was noticeably high only in one animal in the control group (Fig 1B), while it was remarkably high in 5 out of 8 animals in the PS group (Fig 1C), and in all 8 animals in the NS group (Fig 1D).

**Table 2:** Comparison among the three groups in corticosterone levels on GDs 11 and 17.

Corticosterone levels (ng/ml)	*Between groups’ p-values			**Significant main effects			
	Control and PS	Control and NS	PS and NS	F	p	η^2^	Power
GD11	0.967	0.414	0.385	1.628	0.220	0.134	0.305
GD17	**0.010**	**0.001**	0.378	9.875	**0.001**	0.485	0.967
Delta corticosterone	**0.006**	**0.002**	0.356	9.687	**0.001**	0.480	0.964

GD: gestational day, η^2^ = estimates of effect size, NS: noise stress, PS: physical stress.

*The “between groups’ p-values” show p-values for the between group comparisons.

**The “significant main effects” indicate the statistical results of a significant main effect for every measure.

### Morris water task (MWT)

#### Swim time

**Fig 2:**
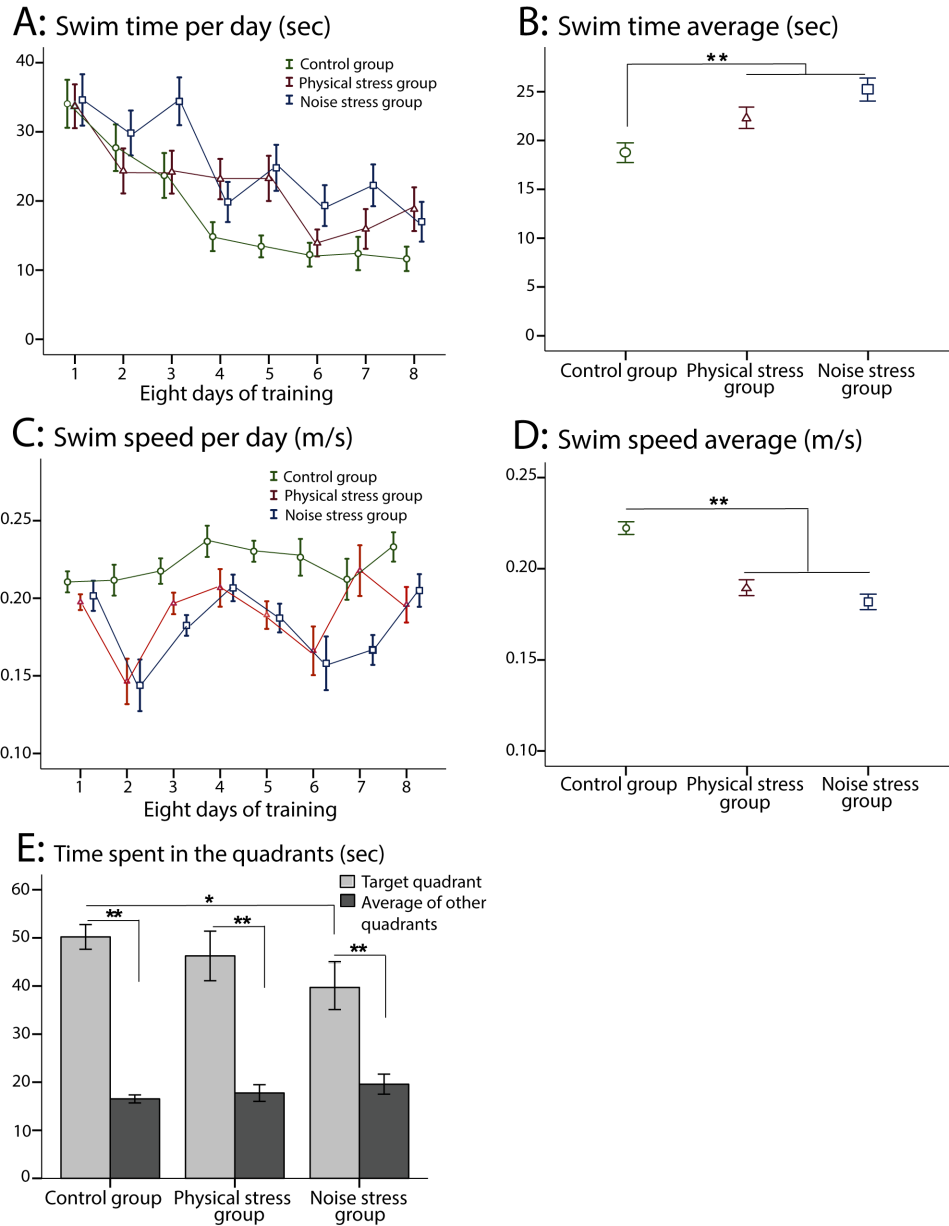
The Morris water task (MWT): A) The swim time (sec) across the 8 days of training in the three groups. B) The swim time average in the three groups (○: control group, Δ: physical stress (PS) group, □: noise stress (NS) group). A significantly higher swim time was observed in both stressed groups relative to the control group. C) The swim speed (m/s) during the 8 days of training in the three groups. D) The swim speed average in the three groups. A significantly lower swim speed was shown in both stressed groups compared with the control group. F) A significant difference was observed between the NS stress group and the control group in probe time (sec). The difference between time spent in the target quadrant and mean of time spent in other quadrants was significant in the three groups. N = 10 in the three groups. Results reported as mean ± S.E.M. Asterisks indicate *p<0.05 or **p<0.01.

Table 3 compares the three groups in swim time (sec), swim speed (m/s), and the probe time (sec) in the MWT. Both stressed groups showed a significantly longer time to reach the platform relative to the control group. Fig 2 illustrates the learning process of the three groups during training days (Fig 2A) and in average (Fig 2B) in swim time. Also in the intra-group analysis, a significant difference was observed in swim time across the 8 days of training in every group (control group: F_7,273_ = 6.289, p = 0.001, partial η^2^ = 0.603, power = 0.997; PS group: F_7,273_ = 4.313, p = 0.002, partial η^2^ = 0.510, power = 0.967; NS group: F_7,273_ = 3.874, p = 0.005, partial η^2^ = 0.520, power = 0.937).

**Table 3:** Comparison among the three groups in swim time, swim speed and probe time in the MWT.

	*Between groups’ p-values			**Significant main effects			
	Control and PS	Control and NS	PS and NS	F	p	η^2^	Power
Swim time (sec)	**0.021**	**<0.001**	0.071	8.421	**<0.001**	0.019	0.965
Swim speed (m/s)	**0.021**	**<0.001**	0.631	8.421	**<0.001**	0.019	0.965
Probe time (sec)	0.463	**0.017**	0.095	3.541	**0.048**	0.260	0.588

MWT: Morris water task, NS: noise stress, PS: physical stress, η^2^ = estimates of effect size.

*The “between groups’ p-values” show p-values for the between group comparisons.

**The “significant main effects” indicate the statistical results of a significant main effect for every measure.

#### Swim speed

Both stressed groups showed a significantly lower swim speed (m/s) to reach the platform compared with the control group (Table 3). Fig 2 indicates the swim speed of the three groups during training days (Fig 2C) and in average (Fig 2D). However, no significant difference was found in swim speed across the 8 days of training in the control group (F_7,273_ = 1.163, p = 0.324, partial η^2^ = 0.028, power = 0.498). Nonetheless, in both stressed groups (PS group: F_7,273_ = 3.646, p = 0.001, partial η^2^ = 0.082, power = 0.974; and NS group: F_7,273_ = 4.014, p <0.001, partial η^2^ = 0.092, power = 0.985), the difference in swim speed across training days was significant.

#### Swim distance

No significant differences were found among the three groups in swim distance (m) (F_2,27_ = 0.341, p = 0.711, partial η^2^ = 0.001, power = 0.105).

#### Probe test

The probe time (sec) was lower in the stressed groups relative to the control group (Fig 2E), and the difference between the NS group and the control group was significant (Table 3). The difference between time spent in the target quadrant and average of time spent in other quadrants was significant in the three groups (control group: t = 12.484, p = 0.001; PS group: t = 5.946, p = 0.001, NS group: t = 7.140, p = 0.001).

#### Correlation between the corticosterone levels and MWT results

A significant positive relationship was observed between the swim time and the delta corticosterone level in the three groups (control group: r = 0.762, p = 0.028; PS group: r = 0.881, p = 0.004; NS group: r = 0.905, p = 0.002). Furthermore, a significant negative correlation was found between the probe time and the delta corticosterone level in the three groups (control group: r = -0.760, p = 0.026; PS group: r = -0.714, p = 0.041; NS group: r = -0.738, p = 0.037).

## Discussion

The main findings of this study were: 1) gestational stress altered spatial learning in mouse dams that were examined 30 days postpartum; 2) the gestational corticosterone level was correlated with the spatial learning and memory function postpartum in all groups; and, 3) loud noise exposure during gestation negatively affected the HPA axis and impaired postpartum memory function as large as or a bit larger than the exposure to physical stressors.

To examine both acquisition and recall of the water task performance, we assessed learning across the 8 test days and recall on day 9 separately. Although a significant improvement in performance was observed in all three groups across training days of the MWT, the stressed groups, an in particular the NS group, had a significantly longer latency to reach the platform relative to the control group. In the probe test, the NS group also spent significantly less time in the target quadrant than the other groups. The results demonstrated an adverse impact of both types of gestational stresses in spatial learning, as well as the negative effect of the noise stress in retention memory in the stressed dams compared with the controls. The findings are congruent with the past studies that reported impairment in spatial learning [56] and retention memory [57], which were correlated with increased gestational plasma corticosterone levels [17, 58], although no previous study explored the spatial learning and memory performance in the postpartum mice mothers who received stress during gestation.

The swim speed of the stressed groups was also significantly slower than the control group. Although the control animals showed a relatively constant speed during the eight days of training, the swim speed in both stressed groups was unstable over the days of training. Previous studies have indicated that regardless of confounding factors such as appetite or differences in body weight, rodents typically swim at similar speeds in the MWT, and changes in swim speed owing to a treatment are typically small. Therefore, a difference in swim speed might not account for a difference in learning the platform location [59]. In addition, in a factor analysis on MWT data in a large number of mice (n = 1500), only latency to escape and probe time were highly related to learning, whereas the swim speed was not significantly related [59, 60]. In our study, however slower swim speed, with marked fluctuations over the days of training, might be related to the impact of the stress procedures on postpartum mental state, attentional skills, or mood as indicated in both human and animal studies [61–63]. This needs to be further explored in the future.

Pregnancy and the postpartum period have been shown as the times of maximal plasticity in both the mother’s brain and behavior. Both short and long term time- dependent changes in cognition and hippocampal neurogenesis, morphology, and electrophysiology have been correlated to pregnancy and mothering [10, 64, 65]. Rat studies have suggested that reproductive experience reduces anxiety throughout pregnancy and the postpartum period and generally enhance cognition, in particular spatial learning and memory [11]. Thus, it has been shown that there is better performance of both primiparous and multiparous females relative to nulliparous females on a dry-land maze at 6, 12, 18, and 24 months of age, long past their last reproductive experience [15], and similarly on the MWT at both 2 weeks and 16 months after weaning [14]. In the later study, whereas a single motherhood experience remarkably enhanced spatial memory and hippocampal synaptic plasticity (LTP) during the entire lifespan of female rats, gestational stress (restraint for 45 min three times each day during last week of pregnancy) totally abolished these positive effects. The authors argued stress-induced hormonal modifications, and in particular increased gestational corticosterone levels, which can impair hippocampal function [17, 18, 66] as one of the possible mediators [14]. The 11þ-hydroxysteroid dehydrogenase-2 (11þ-HSD-2) is an enzyme that is present in the placenta and central nervous system, and converts extra levels of corticosterone to relatively inactive products in normal pregnancies. In mid-gestation, the expression of this enzyme is greatly decreased in rodents [67–69], and it seems that gestational stress can decrease it further, and lead to long-lasting changes in both hippocampal neurogenesis and morphology.

In our study, the corticosterone level was significantly higher on GD17 than GD11 in both stressed groups compared with the control group. The interesting finding was the positive relationship between the delta corticosterone level and the swim time, as well as the negative relation between the delta corticosterone level and the probe time in the three groups. Previous findings indicate the long-lasting dysfunction of the HPA axis under gestational stress [7, 70], as well as elevated corticosterone level in both dams and offspring [17, 70]. Increased corticosterone levels in our stressed mice are likely due to the effect of stress and also the gradual increase of exogenous corticosterone level during the course of pregnancy. As the relationships between delta corticosterone and swim speed/probe time were significant for both control and stressed groups, we argue that a greater increase in corticosterone levels across pregnancy predicts greater swim time and worse performance for the probe test, even when testing is performed weeks later.

There are some caveats of the study that should be considered. Although we used well-known protocols for exposing animals to NS and PS, these types of experimental stressors are different to some extent than those typical auditory and physical stress observed in natural environments. Thus, experimental conditions that provide more analogous models of stress to natural stressors are recommended for future studies. We also did not measure corticosterone at the time of spatial learning, which in hindsight would have provided some insight into the HPA activity at the time of the learning. In addition, including other female groups comprising a non-pregnant stressed group as well as a non-pregnant non-stress group might provide further information to shed light more on the effect of reproductive experience in the corticosterone alternations as well as the memory performance. Research into the neural mechanisms underlying cognitive changes owing to gestational stress in the rodent postpartum period is only beginning. It is clear that studies on hippocampal morphology and neurogenesis are needed to determine how pregnant mice exposed to gestational stress exhibit reactions to corticosterone fluctuations during pregnancy, early postpartum, lactation, weaning, and months later.

## Conclusion

This study was novel in using “noise stress” with pregnant mice to investigate the effect of stress during pregnancy on spatial learning and memory performance in the postpartum dams. The results suggested an adverse effect of both gestational stresses, particularly noise exposure, on gestational corticosterone levels as well as postpartum learning and memory function relative to the controls. The strong relation between the MWT results and the delta corticosterone levels demonstrated the lasting effect of stress during gestation on postpartum memory performance. The relatively large effects of loud noise exposure during gestation demonstrate the importance of examining the role of environmental noise on pregnant mothers.
